# A Comprehensive Review on Preparation, Evaluation and Applications of Deformable Liposomes

**DOI:** 10.22037/ijpr.2020.112878.13997

**Published:** 2021

**Authors:** Devika Nayak, Vamshi Krishna Tippavajhala

**Affiliations:** *Department of Pharmaceutics, Manipal College of Pharmaceutical Sciences, Manipal Academy of Higher Education, Manipal, Karnataka, India.*

**Keywords:** Deformable liposomes, Elastic liposomes, Flexible liposomes, Transdermal drug delivery, Skin penetration

## Abstract

Elastic or deformable liposomes are phospholipid-based vesicular drug delivery systems that help improve the delivery of therapeutic agents through the intact skin membrane due to their deformable characteristics that overcome the problems of conventional liposomes. In the present review, different types of deformable liposomes such as transfersomes, ethosomes, menthosomes, invasomes and transethosome are studied, and their mechanism of action, characterization, preparation methods, and applications in pharmaceutical technology through topical, transdermal, nasal and oral routes for effective drug delivery are compared for their potential transdermal delivery of poorly permeable drugs. Due to the deformable characteristics of these vehicles, it resulted in modulation of increased drug encapsulation efficiency, permeation and penetration of the drug into or through the skin membrane and are found to be more effective than conventional drug delivery systems. So deformable liposomes can, therefore, be considered as a promising way of delivering the drugs transdermally.

## Introduction

For the transfer of drugs through topical and transdermal routes, the three main key targets are found to be on the surface of the skin, epidermis, dermis and subcutaneous or systemic circulation. When the disease is present within the target organs, delivering or targeting the drugs to the different layers of the skin becomes more relevant, like microbial infections and fungal diseases. The topical and transdermal route is more effective as it reduces the adverse effects, bypasses the first-pass metabolism, and is one of the alternative modes of therapy to systemic, oral, and other administration routes (1).

*Liposomes*

Alec Bangham made the first description of liposomes in 1964 as closed vesicles made from phospholipids, shaped in excess water. Liposomes are vesicular systems with an aqueous internal environment bounded by the phospholipid bilayer formed when the phospholipids are dispersed into water (2). It is from the combination of two Greek words, “lipo” meaning fat and “soma”-meaning body (3). Liposomes are spherical artificial vesicles that can be created from non-toxic natural phospholipids and cholesterol. They consist of aqueous units surrounded by one or more bilayers of lipid, where the head groups of the polar regions are oriented in the interior pathway and exterior aqueous phases. It can entrap both lipophilic and hydrophilic drugs in its liposomal system. Liposomal vesicles have a rigid structure, wide size, >400 nm in diameter (4). Liposomes can supply a wide range of hydrophilic drugs (such as carboxyfluorescein, sodium fluorescein), lipophilic drugs (such as tretinoin, retinoic acid), skin proteins and macromolecules (5). Liposomes are being used for transdermal and topical delivery of many drugs and, systemic treatment of local diseases (6).

*Deformable liposomes*

**Figure 1:**
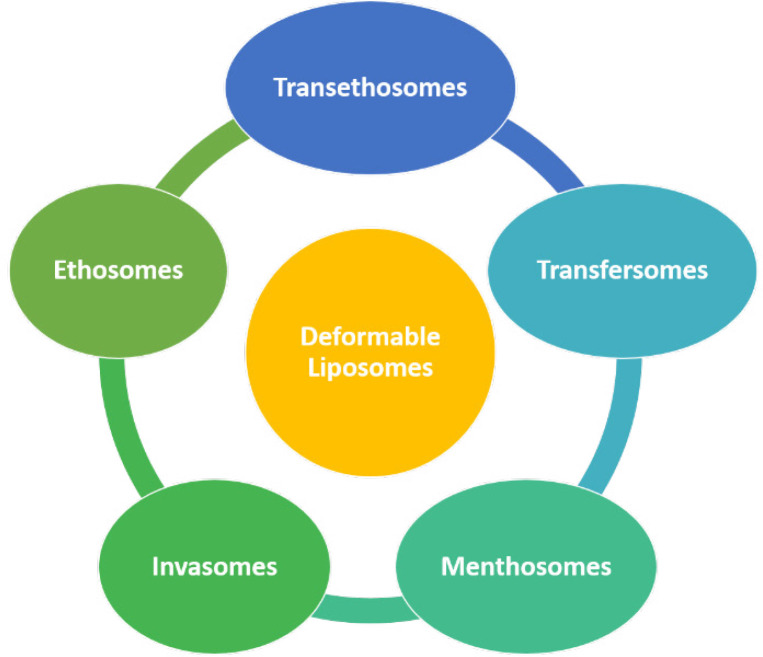
Schematic representation of different deformable Liposomes

In the early 1990s, due to the poor penetration of drugs through the skin, Cevc and Blume modified the bilayer structure of liposomes by adding of edge activators to the liposome composition, and the resultant modified liposomes were called “Deformable Liposomes (7)”. Deformable liposomes are known by different names – flexible liposomes, elastic liposomes, ultra-deformable liposomes, ultra-flexible liposome**s **(8)**. **These are bilayer biocompatible vesicular drug delivery systems used for many drugs for their biochemical, cosmetic and therapeutic purposes. The first group consists of deformable vesicles: such as transfersomes and nonionic surfactants-based flexible vesicles. It is obtained by mixing with certain hydrophilic solutes or by adding edge activators into bilayers. This group of vesicles acts as carriers for the drugs that penetrate into intact skin hydrophilically. The second group comprised penetration enhancer-including vesicles, invasomes, and ethosomes (9). Various deformable liposomes are depicted in Figure 1.

“-Deformable liposomes”-namely transfersomes, ethosomes, menthosomes, invasomes, and transethosomes, offer several advantages over the conventional drug delivery system, as described below.

High stress-dependent adaptability (high elasticity) in deformable liposomes, whereas stiffness of the bilayer is seen in conventional liposomes. It is mainly due to the dissimilarity in the structure between deformable liposomes (made from phospholipids with cholesterol or without it) as well as traditional liposomes (11).

Deformable liposomes contain smaller vesicle sizes and higher elasticity because of the presence of edge activator.

Compared to conventional liposomes, deformable vesicles have higher entrapment efficiency and higher potential in skin permeation. 

High elasticity and membrane hydrophilicity promote vesicles to avoid fusion and aggregation due to osmotic stress that makes it difficult for the conventional liposomes.

Deformable liposomes show greater hydrophilicity-allowing more swelling of the elastic membrane than conventional liposomes (12).

Following the osmotic gradient, deformable liposomes penetrate deeper epidermis layers through lipid lamellar regions of the stratum corneum, In contrast, conventional liposomes merge with the lipids of the skin, dehydrate and remain near the skin surface (4).

**Deformable Liposomes**

*Transfersomes*

The first generation of elastic liposomes are transfersomes or ultra-flexible vesicles consisting of one aqueous internal compartment surrounded by lipid bilayers (13). Transfersomes were obtained by adding edge activators (EA) into lipid bilayers. It is a single surfactant chain (sodium deoxycholate, spans, and tweens) with a high curvature radius that increases the flexibility and elasticity of the lipid bilayers, which makes ultra- deformable vesicles (4, 14). 

**Preparation method**

Materials widely used in the formulation of transfersomes are various phospholipids, surfactants, alcohol, dye, buffering agent, *etc*. Different additives used in the formulation of transfersomes are summarized in Table 1. Transfersomes are generally prepared using the thin-film hydration method. In this method, accurately weighed phospholipids, drug, and edge activator quantities are dissolved in a mixture of chloroform: methanol. By rotatory evaporation, under reduced pressure above the transition temperature of the lipids, the organic solvent was evaporated to form a lipid film on the flask wall. The final solvent residues are removed overnight under a vacuum. The formed thin, dry lipid film is hydrated with saline phosphate buffer. The lipid vesicle was then allowed to swell at room temperature, and the resulting multilayer vesicle was probe sonicated to reduce the size of the vesicle and stored at 4 ^o^C (15, 16).

**Mechanism of action**

**Figure 2:**
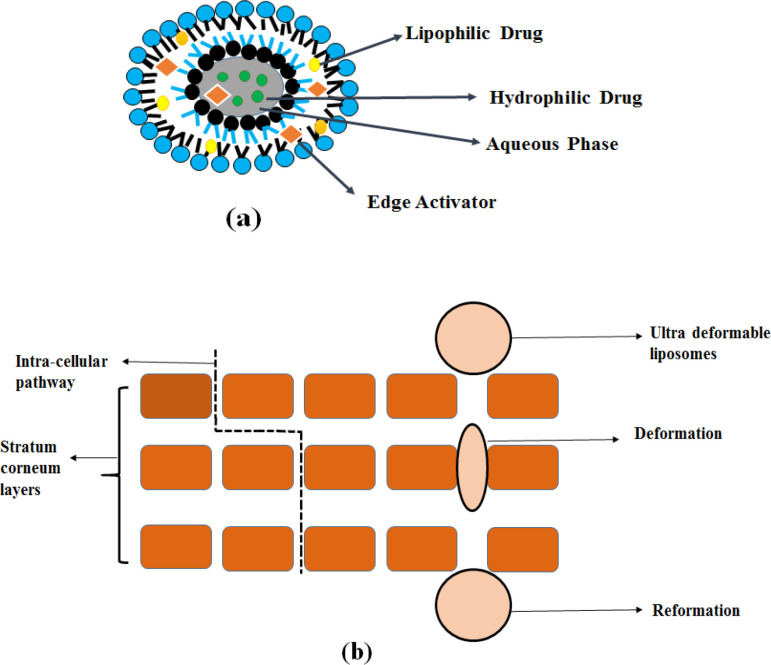
Transfersome (a) Structure and (b) Mechanism of action

The epidermal surface contains low water content by nearly 15%, less than other skin layers**.** Due to the difference in water content, a hydration gradient exists between them. Transepidermal water concentration is narrow which extends over outer skin layers (17). Elastic drug carriers spontaneously cross the intact skin under the influence of a strong, naturally occurring transcutaneous moisture gradient (18). Transfersomes overcomes the difficulty of skin penetration by compressing the stratum corneum intracellular sealing lipid (19). Bilayer deformability can be achieved by the presence of a single-chain surfactant (edge activator) with a high curvature radius which impairs the stability of the bilayer membrane. The flexibility of membrane transfersomes is controlled by the proper mixing of phospholipids with surface-active agents. The resulting elasticity of the transfersomes membrane reduces the risk of complete rupture of the vesicles in the skin. It enables transfersomes to follow the natural water gradient throughout the epidermis when applied under non-occlusive conditions (20). It has been depicted in Figure 2.

**Applications of transfersomes**

*Delivery of insulin*

To overcome the difficulty in improving the transport efficiency of large molecular weight peptide-like insulin-, and to limit their drawbacks like high cost, physical stability, and deposition of the fat in the injection sites, transfersomes as a vesicular system was used for the transdermal insulin delivery. It is one of the successful ways to deliver large molecular weight drugs through the transdermal route. Transfersomal suspension of insulin was prepared and incorporated into methylcellulose gel so the resultant transfersomal insulin gel which overcomes the problems of conventional insulin delivery. With the influence of current supply, *i.e.,* Iontophoresis, an optimized transfersomal gel of insulin showed maximum permeation flux than the normal condition. So the optimized and developed transfersomal gel of insulin can reduce the high blood glucose levels and be transdermally administered in the treatment of insulin-dependent diabetes mellitus (21).

*Delivery of Corticosteroids*

Transfersomes are used for the transdermal delivery of corticosteroids (hydrocortisone and dexamethasone). Transfersome corticosteroids are biologically active at doses several times lower than those currently used in dermatic formulations to treat skin diseases. Application of transfersomal corticosteroids fastens the action, onset of anti-edema effects and bioactivity without affecting the mechanical abrasion. This application of new deformable liposomes by incorporating corticosteroids allows a new skin diseases method. (22). 

*Delivery of non-steroidal anti-inflammatory drugs (NSAIDs)*

Transdermal skin delivery can overcome the side effects of the gastrointestinal tract such as indigestion and stomache by using novel deformable liposomes such as transfersomes. As per this study, meloxicam-loaded cationic transfersomes were prepared by the sonication method. The fusion of the drug into cationic transfersomes resulted in a reduction of particle size,- and high entrapment efficiency and enhanced the skin permeation through vesicle adsorption and by fusion with stratum corneum. It was found to be better than conventional liposomes and suspension (23). 

*Delivery of anticancer agents*

Novel deformable liposomes, *i.e., *transfersomes are used for the transdermal delivery of anticancer agents like 5-fluorouracil. It overcomes the drawbacks of conventional creams like poor drug penetration into the deeper parts of the tumor. It was prepared by the rotatory evaporation sonication method and the optimized formulation was converted into the gel using_ €_Carbopol^®^ as a polymer. It enhanced the *in-vitro* skin permeation and deposition of the drug to the deeper parts of the skin and was better than the marketed preparation (24).

*Delivery of vitamin A derivatives*

Retinoids have important effects on skin cells. Retinoid levels in the skin are involved in the correct cellular maturation of keratinocytes. Alterations in retinoids levels produce a destruct of coenocytes and, - consequently, increase transepidermal water loss. Pena-Rodríguez *et al. *developed retinyl palmitate- loaded transfersomes (RPLT) for effective epidermal delivery through the skin. It is prepared by the sonication method and incorporated into a cream formulation and was subjected to various physicochemical characterization, diffusion and skin penetration assays. 

*An in-vitro *study of RPLT showed improved penetration through the skin’s stratum corneum layer than free retinyl palmitate control formulation. A fluorescent microscopy experiment was performed to validate the increase in the epidermal delivery of retinyl palmitate in transfersomes. The result showed a significant increase in skin penetration of the retinyl palmitate when formulated into transfersomes with an accumulation of active ingredients in epidermis and dermis layers of the skin. The *in-vivo* study analysis demonstrated the compatibility of the transfersomes formulation and was a successful candidate for the effective delivery of highly lipophilic drugs (25).

*Delivery of Local Anesthetics*

Most of the available local anesthetics have short term pain relief, but it needs frequent administration to obtain the long-term duration of action. To provide a sustained release formulation irrespective of its dose and route of administration, Bnyan and Khan *et al. *formulated novel deformable liposomes as sustained-release transfersomes containing local anesthetics. Taguchi design experiment software was used to optimize the parameters related to transfersome formulation and it was prepared by a thin-film hydration technique. HPLC method for lidocaine was developed and validated and valid for the result of analysis as per ICH guidelines. *In-vitro *release study of optimized lidocaine-transfersome formulation showed sustained delivery of drugs compared to free drugs (26).

**Ethosomes**

Elastic nanovesicles based on phospholipids,- containing a high percentage of ethanol are known as ethosomes (20-45%) (27). Compared to conventional liposomes, it is more efficient in the deeper delivery of the substances into the skin. Ethosomes containing a high percentage of ethanol acts as an enhancer for permeation, and it is added to prepare the elastic nanovesicles (27). Ethosomes can entrap either hydrophilic, lipophilic, amphiphilic drug molecules with various physicochemical characteristics (28).

**Preparation methods**

Materials that are widely used in the formulation of ethosomes are various phospholipids, surfactants, alcohol, vehicle, cholesterol, and propylene glycol as summarized in Table 1.

*Mechanical dispersion method*

Ethosomes were prepared by the mechanical dispersion method. In a dried, round-bottomed flask (RBF), phospholipids at different concentrations were dissolved in a chloroform-methanol mixture. The organic solvent was then evaporated using rotavapor,-by maintaining above the transition temperature of lipids. A thin film of lipid was then formed on the RBF wall and hydrated using different concentrations of a hydro ethanol mixture containing the drug. The preparations were subjected to sonication to obtain nanosized ethosomes (29).

*Hot method*

The drug was dissolved in a mixture of propylene glycol: ethanol and added to phospholipids dispersions of water on a magnetic stirrer and the temperature was maintained at 40 °C. The mixture was then probe sonicated and then homogenized using a high-pressure homogenizer to get ethosomes (28). 

*Cold method*

In a conical flask, soya lecithin was dissolved in ethanol, and then it was kept for magnetic stirring. During the stirring, a small quantity of propylene glycol was added to this alcoholic solution and the temperature of this solution was maintained at 30 °C. The drug was dissolved in an aqueous phase containing distilled water, heated separately, and maintained the same temperature as before (alcoholic solution). To the alcoholic solution, the aqueous phase was then added slowly using a mechanical stirrer in a closed vessel. Then the mixture was probe sonicated to get ethosomes (30). 

**Mechanism of action**

Absorption of the drug is explained in two pathways: The ethanol effect and the Ethosomes effect.

*Ethanol effect*

Ethanol interacts with polar head groups of lipid molecules, thereby decreasing the stratum corneum melting point and increasing the membrane permeability and fluidity. It also acts by pull and push mechanisms. Due to an increase in thermodynamic activity with the stratum corneum, ethanol evaporates from the vesicular system resulting in a push effect, it enhances the penetration and permeability of the vesicles resulting in a pull effect. So the soft and flexible vesicles penetrate deeper skin layers, thus ethanol works as an effective enhancer in penetration (27, 29 and 31). 

*Ethosomes effect*

The presence of ethanol in the ethosomes results in increased fluidity of lipids and permeability of the cell membrane, so ethosomes fuses with the skin lipids and penetrates to the deeper layer of the skin and releases the drug (32).

**Figure 3:**
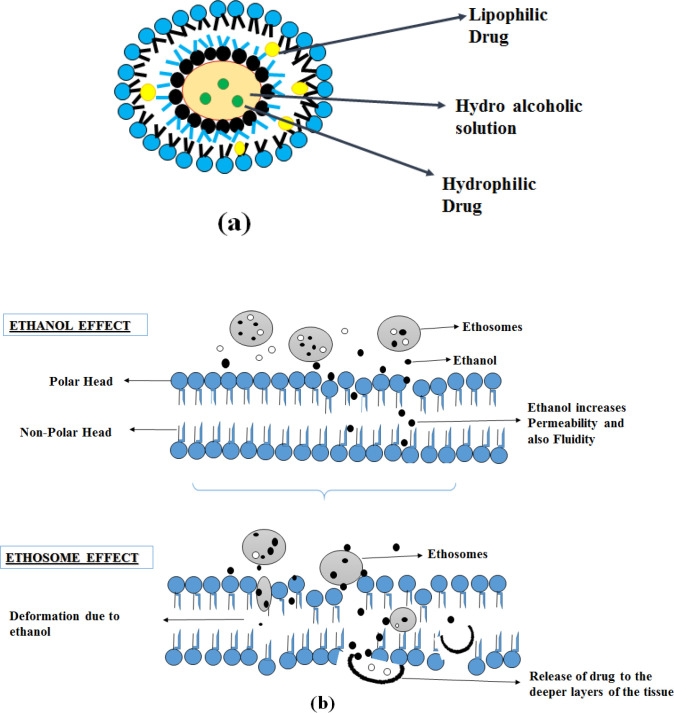
Ethosome (a) Structure and (b) Mechanism of action

These mechanisms of action, along with the structure described in Figure 3.

**Applications of Ethosomes**

*Antifungal drug delivery*

Faisal *et al.* used ethosomes as a vesicular carrier for the topical delivery of voriconazole to treat fungal infection. To reduce the side effects of oral and parenteral therapy, to increase the efficacy and the topical skin delivery of the drug, voriconazole encapsulated ethosomal preparation was used. Voriconazole ethosomes were prepared by the cold method and were subjected for evaluation to determine the effect of the formulation on the vesicle properties and antifungal efficiency. It showed increased entrapment efficiency and *ex-vivo *permeability through abdominal rat skin. The particle size was uniform and in the compared with the hydroalcoholic drug solution. *In-vitro* antifungal activity of the optimal ethosomal preparation was tested against *Aspergillus flavus*. It showed that the developed ethosomal preparation of the voriconazole was successful for treating the fungal infection (33).

*Delivery of NSAIDs*

To limit the drawbacks associated with the GI tract, transdermal ethosomes are used for the delivery of ibuprofen through the skin. It was tested for antipyretic and *in-vivo *effects in rats. Results of antipyretic effects in rats showed a decrease in the body temperature in fevered animal models and reached normal body temperature. It showed an enhanced duration and fastened the antipyretic effects on the transdermal application of ibuprofen ethosomes gel and lasted for 12 h as compared with the oral treatment (34). 

*Delivery of anti-hypertensive drugs*

Valsartan has low bioavailability and poor absorption through the gastrointestinal tract, so ethosomes are used as a carrier for transdermal delivery. The *in-vivo *study was carried out in hypertensive rats by inducing methylprednisolone acetate in Wistar albino rats. The results showed a better and prolonged anti-hypertensive effect in valsartan-treated transdermal ethosomes than oral valsartan suspension (35). 

*Delivery of testosterone*

Oral testosterone delivery is associated with liver metabolism, low bioavailability, and dose-related side effects. Transdermal ethosomes delivery of testosterone was considered of the efficient and convenient ways to deliver the testosterone and bypasses its metabolism and reduces its side effects. *The* *In-vivo *and* in-vitro *study results showed enhanced bioavailability and skin permeation in the ethosomal formulation loaded with the testosterone over the marketed transdermal patch (36). 

*Delivery of Anticancer agent*

The main biologically active complex of black cumin seed, *i.e.*, thymoquinone, has demonstrated anticancer activity against several tumors. To obtain a constant release of drugs in plasma and to avoid the frequent dosing of a drug regimen, Nasri *et al.* considered the transdermal route as an effective means of therapy for the delivery of thymoquinone through a novel deformable vesicle, *i.e.,* ethosomes. Response surface design was used for the preparation of ethosomes *i.e.,* encapsulation of thymoquinone. The central composite design was used to study the effects of different parameters like phospholipid, cholesterol and ethanol concentration. The results of the *in-vitro* study showed enhanced skin permeation and deposition (37).

*Treatment of Gout*

FXT is a xanthine oxidase inhibitor drug, which is having poor bioavailability and dissolution rate. So, El-Shenawy *et al.* developed ethosomal formulations of febuxostat by the cold method. The developed ethosomal formulation was converted into a gel and subjected to various physicochemical and *ex-vivo* studies. The optimized formulation showed smaller particle size and high entrapment efficiency with maximum stability. *The ex-vivo* study revealed that the formulated gel showed maximum skin penetration and was successful in the treatment of gout, and was an alternative mode of therapy other than oral administration of tablets (38).

**Transethosomes**

“-Transethosomes (TELs)-“, contains a high content of ethanol together with permeation enhancer or with an edge activator which is having the property of both transfersomes and ethosome (39). Transethosomal drug delivery system was found to be more effective than other conventional vesicular drug delivery systems. (*i.e.*, liposomes, transfersomes and ethosomes) in terms of responses such as entrapment efficiency, degree of deformability, skin retention and permeation of the drug (40). Due to the combination of both edge activator and ethanol, transethosomes cause rearrangement of lipid bilayers of the vesicles. These vesicular systems are more deformable than conventional drug delivery systems allowing higher membrane passage and are quite easy to scale up (41).

**Preparation methods**

Materials which are widely used in the formulation of ethosomes are various phospholipid, surfactants, alcohol, permeation enhancer, and buffers as summarized in Table 1. 

*Thin-film hydration method*

The Edge activator, drug, and lipid were weighed accurately and dissolved in a mixture of chloroform and methanol. A thin film was formed on the evaporation of this solution using a rotary evaporator under reduced pressure. The thin film formed was hydrated using pH 7.4 (saline phosphate buffer) with varying ethanol concentration, and the prepared suspension was kept overnight for complete hydration (40). 

*Cold method*

In a conical flask, soya lecithin was dissolved in ethanol, and then it was kept for magnetic stirring. Edge activator was added to this alcoholic solution during stirring, and the temperature of this solution was maintained at 30 °C. The drug was dissolved in an aqueous phase containing distilled water, heated separately, and maintained the same temperature as before (alcoholic solution). To the alcoholic solution, the aqueous phase was then added slowly using a mechanical stirrer in a closed vessel. Then the mixture was probe sonicated to get nanosized Ethosomes (42).

*Ethanol injection method*

The organic phase was obtained by dissolving phospholipid, drug, and surfactants in ethanol under mixing and the temperature was maintained at 35 ± 2°C. The aqueous phase containing edge activator and water was added to the organic phase in a small stream with continuous stirring. To avoid ethanol evaporation, the resultant ethosomes solution was sealed using a glass vial under the protection of the nitrogen atmosphere (43).

**Mechanism of action**

**Figure 4:**
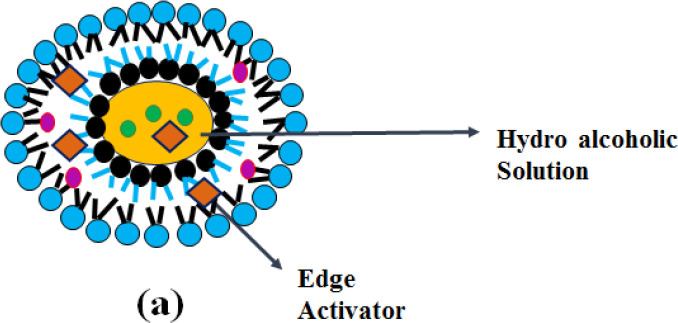
Structure of Transethosome

“-Transethosomes (TELs)-“, which is derived from ethosomes and transfersomes, contain an edge activator and with a high ethanol content that acts as an enhancer of permeation. As shown in Figure 4, the synergistic mechanism between surfactant, ethanol and skin lipids leads to increased transethosomal penetration of the skin. Phospholipids, together with ethanol interact with the stratum corneum and loosen the intercellular lipid structure and increase the skin partitioning of the drug and lead to enhancement of drug penetration. The presence of ethanol allows them to penetrate the deeper skin layers. Permeation enhancer’-s decrease the phase transition temperature of the skin lipids by intercalating between the liposomal bilayer and increasing the fluidity (39).

**Applications of Transethosomes**

*Delivery of NSAIDs*

Transethosomes are novel elastic vesicular carriers having the advantage of both transfersomes and ethosomes. To overcome the gastrointestinal side effects transethosomes are considered for the transdermal delivery of NSAIDs. Transethosomes of ketorolac tromethamine were prepared by the cold method incorporated into the Carbopol^®^ ultrez 10 polymers. *The* *in-vitro *diffusion, *ex-vivo *skin permeation, and deposition study showed a higher skin permeation profile in transethosomal gel compared to hydroethanolic and plain drug solution. After applying transethosomes encapsulated ketorolac tromethamine gel in inflammation-induced rats; *the* *in-vivo* study showed inhibition of edema and swelling than marketed gel (42). 

*Treatment of Gout*

To overcome the side effects, poor bioavailability of colchicine through the oral route, transethosomes are used as a potential carrier for transdermal delivery of colchicine. The optimized transethosomal loaded colchicine gel was characterized. It was noted that the amount of colchicine permeated (after 24 h) through the skin of rats from the transethosomal gel was significantly higher than the NE gel. These carriers provided an alternative route for drug administration that overcomes the side effects of poor bioavailability (44). 

*Delivery of Antifungal drugs*

Transethosomes, a novel vesicular carrier was used for the transdermal delivery of voriconazole. Transethosomal formulation showed higher elasticity, permeability and skin deposition due to the synergistic effect of ethanol in conjunction with ethanol, edge activator or permeation enhancer and it was found to be more effective when compared with deformable liposomes and conventional liposomes (39).

*Delivery of Anti-hypertensive drug*

Propranolol hydrochloride has 23% of bioavailability when administered through oral route due to hepatic first-pass metabolism and degradation of drugs by GI enzymes. The transdermal route of drug delivery is considered one of the effective delivery paths for transferring the drugs, as it avoids the GI side effects. Kumar *et al.* prepared transethosomes loaded propranolol hydrochloride by homogenization method and converted into the gel using carbopol^® ^934 polymers. *In-vitro* drug release study showed sustained release of drug with maximum stability up to 5 months at a temperature of 25 ± 1.5 °C, 4 ± 1.5 °C with 75% relative humidity in the dark environment. *In-vitro* skin permeation study was performed through the abdominal skin of male Sprague Dawley rat and the developed transethosomal gel showed deeper penetration of the drug through the skin. Plasma concentration study revealed that the transethosomal gel maintained an effective drug plasma concentration than the marketed oral tablets. So, it can be considered a promising carrier for delivering non-selective β-blocker drugs transdermally (45).

**Menthosomes**

Menthosomes are the novel ultra-deformable carrier consists of menthol, Phospholipid and edge activator containing cationic surfactants (*e.g.-, *cetylpyridinium chloride) (46). L-Menthol provides enhanced skin penetration to facilitate transdermal drug delivery of various drugs by increasing drug partitioning and diffusion (47). By affecting the lamellar lipid bilayer structure menthol and cationic surfactants increase the fluidity and enhance the transdermal drug delivery (46).

**Preparation method**

Materials that are widely used in the formulation of menthosomes are edge activators (most commonly used is cationic surfactants), alcohol, a permeation enhancer, buffers, and cholesterol. These are summarized in Table 1. Menthosomes are generally prepared by the sonication method. Lipid component mixtures containing phospholipid, cationic surfactants, drug, and cholesterol are dissolved in the chloroform-methanol mixture, - and mixed. On evaporation of the solvent under a stream of nitrogen gas, a thin film of lipid is formed, -To remove the residual solvent, it was then placed in a desiccator. The dried thin film of lipid was then hydrated with 0.01 M acetate buffer, and the formed vesicle was then bath sonicated to reduce the size of the vesicles and stored at 4 °C in an airtight container (48).

**Mechanism of action**

**Figure 5:**
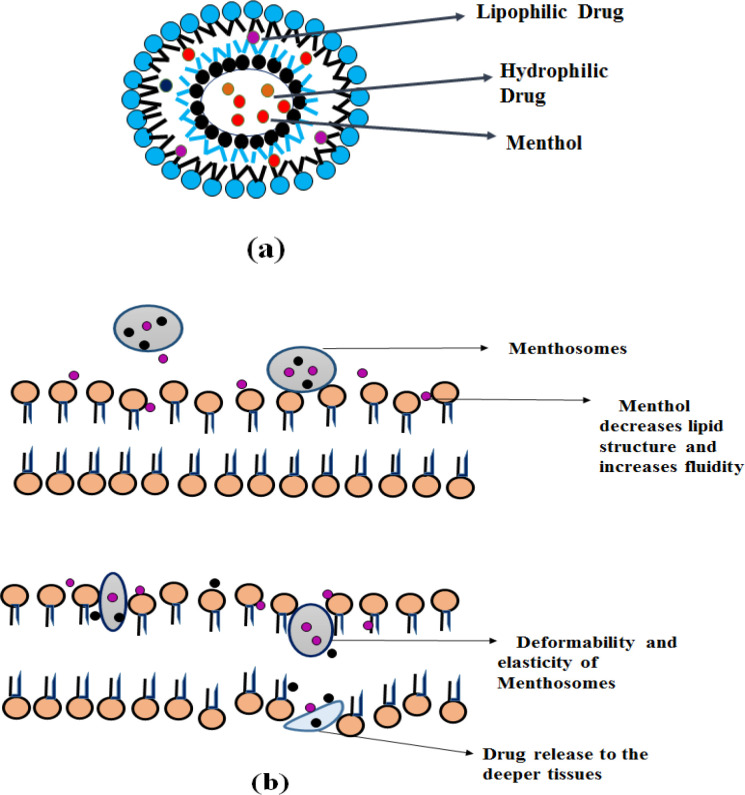
Menthosome (a) Structure and (b) Mechanism of action

The stratum corneum lipid structure consists of hexagonal and orthorhombic hydrocarbon chain packaging. Menthol decreases the value of hexagonal/orthorhombic hydrocarbon chain packing (RH/O) and orderly arranged lipid microstructure, thereby increases the stratum corneum intercellular lipid fluidity. Thus promotes transdermal drug penetration. The presence of cationic surfactants in menthosomes increases the deformability and elasticity by destabilizing the bilayer and acts as a solubilizing agent. Cholesterol decreases the packing density and rigidity of the PC molecule (30, 46 and 47). It is depicted in Figure 5.

**Application of Menthosomes**

*Delivery of NSAIDs*

Due to the adverse effects of the GI tract and poor water solubility when administered orally, it was considered that transdermal delivery was an appropriate approach for meloxicam delivery. Novel meloxicam menthosomes formulation significantly improved penetration of meloxicam through the skin, elasticity, flux, and deposition of the drug compared to meloxicam suspension, transfersomes, and conventional liposomes (48).

**Invasomes**

Invasomes are vesicular drug delivery systems based on phospholipid containing ethanol and terpenes, which deform the vesicle and enhance penetration (49). Invasomes vesicular system enhances the penetration of both (lipophilic and hydrophilic) drugs through the skin. 

**Mechanism of Action**

**Figure 6:**
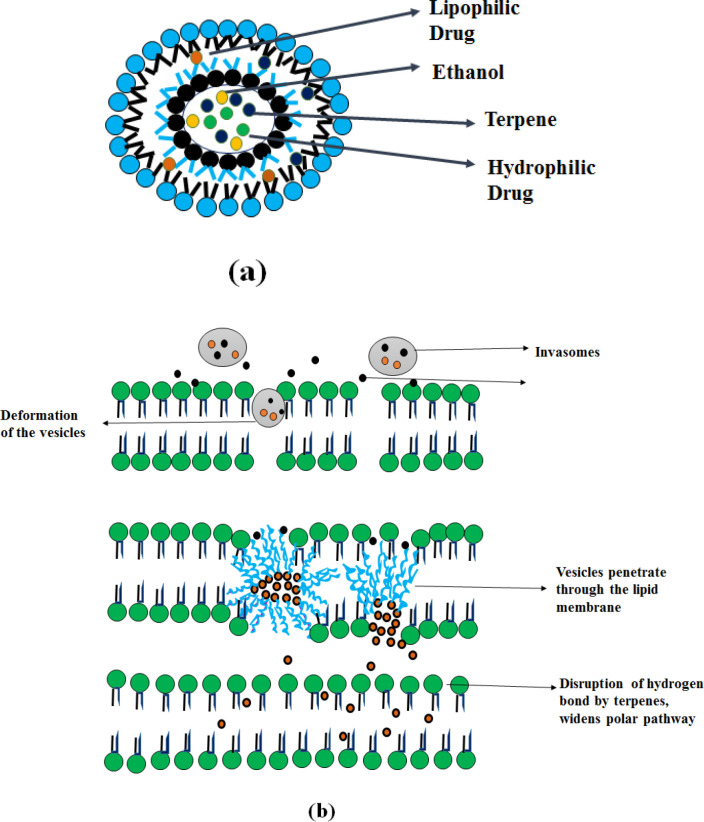
Invasome (a) Structure and (b) Mechanism of action

As shown in Figure 6, the presence of terpenes in the invasomes (constituents of volatile oils) disturbs the tight packing of the stratum corneum (50). This provides an enhancement in skin permeation to facilitate transdermal drug delivery. It can contain a mixture of terpenes like citral, limonene, cineole, or a single terpene (citral). Terpenes consist of a repeated unit of isoprene (C_5_H_8_), combined from head to tail, classified according to the number of isoprene units and by chemical groups (esters, ketones) (51). 

**Preparation methods**

Materials which are widely used in the formulation of ethosomes are various phospholipid. Ethanol and terpenes are summarized in Table 1. Following methods are mainly used in their preparation.

*Thin layer evaporation technique*

Phospholipid, drug, and terpene are dissolved in a mixture of methanol: chloroform (1:2 v/v) in a dry, clean RBF. The organic solvent was then removed by the rotatory evaporator and the remaining traces of solvent are removed overnight under vacuum. The thin lipid film formed on the bottom of the RBF was hydrated at room temperature with an ethanolic-phosphate buffer saline mixture of 7.4 pH (60 rpm). Then the lipid vesicles were allowed to swell at room temperature. The sizes of resultant multilamellar vesicles reduced by probe sonication. (52).

*Vortexing method*

The mixture of terpenes (1% w/v) and the drug was dissolved in a phospholipid ethanolic mixture. The phospholipid ethanolic mixture was vortexed and then sonicated for the desired period to obtain a clear solution. To this solution, phosphate buffer saline was added under constant swirling with a syringe. The resultant multilamellar vesicle was extruded with polycarbonate membrane filters of various pore sizes (49).

**Applications of Invasomes**

*Delivery of anti-hypertensive drugs*

 To enhance the poor bioavailability and bypass the first-pass metabolism, Qadri *et al.* prepared isradipine loaded invasomal transgel (ILIT) andassessed its physicochemical characteristics *in-vivo* pharmacodynamics study. Isradipine is an effective calcium channel blocker used in the management of hypertension. *In-vitro*, the transdermal penetration study showed maximum transdermal penetration flux because of the presence of both ethanol and terpenes in the invasomes which makes the particle more deformable and acts as a penetration enhancer. The optimized formulation was incorporated into carbapol^®^ 943 polymer and the resultant invasomal gel were subjected for *in-vivo *pharmacodynamics study. It showed a reduction of blood pressure in deoxycorticosterone acetate induced hypertensive rats after the application of ILIT (52). 

*Treatment of Acne*

Dapsone (DPS) is a compound of sulphones with a unique combination of antibacterial and anti-inflammatory effects. After oral administration, it is fully absorbed and results in 85% bioavailability. Despite its desirable pharmacological activities, methemoglobinemia and hemolytic anemia are the serious side effects associated with DPS. Therefore, topical treatment with novel vesicles was considered to reduce systemic exposure. Invasomes improve penetration of drugs percutaneously and are compared to conventional liposomes. It was prepared using a method of thin-film hydration and optimized with full factorial design. The *in-vivo* study showed higher skin deposition in invasome loaded dapsone than alcoholic drug solution (53). 

*Delivery of herbal formulation*

Cyclodextrin and hydroxyl propyl ß cyclodextrin were used to enhance the bioavailability and poor solubility of curcumin. These complex mixtures are incorporated into invasomes and then converted into a gel for transdermal delivery. The formulation containing terpenes showed increased permeation in the skin than conventional liposomes due to the combined effect of terpenes and ethanol and showed no signs of edema on the rabbit’s skin (54).

*Treatment of Erectile Dysfunction*

Avanafil is a non-selective phosphodiesterase inhibitor used for treating erectile dysfunction. It suffers from several disadvantages like rapid absorption of the drug upon oral administration and systemic metabolism. Transdermal delivery of the drug is considered an effective drug delivery route as it reduces the side effects and provides controlled drug delivery. The bioavailability of avanafil is improved by Ahmed et al. by formulating into novel vesicles i.e., Invasomes. Box-benkhen design was used to optimize the formulation parameters like vesicle size and entrapment efficiency. The developed and optimized invasomes were converted to transdermal film and subjected to *in-vivo* and *ex-vivo *-investigations. The percentage of terpene in invasomes significantly affected to decrease the particle size and to provide maximum entrapment efficiency. *Ex-vivo *-skin penetration study demonstrated maximum skin penetration from the hydroxypropyl-methyl cellulose-based transdermal film containing avanafil loaded invasome in comparison with that of controlled avanafil preparation. It was found to be a good candidate for the transdermal delivery of drugs through the skin (55).

The applications of different deformable liposomes are summarized in Table 2.

Table 1 summarizes the formulation components of these various types of deformable liposomes along with the examples and their uses.

Various applications of these deformable liposomes are summarized in Table 2 as follows:

**Characterization study of deformable liposomes**

*Size of the particle and surface charge*

Particle dimension, and charge distribution of deformable vesicles, can be determined using laser scattering particle size distribution analyzer, zeta potential analyzer and laser scattering particle size distribution (57, 58).

*Transmission electron microscopy (TEM)*

Visualization of the vesicles was carried out using TEM. Sample for TEM is prepared using conventional negative-staining methods using 1% aqueous PTA (Phosphotungstic acid). For staining on a carbon-coated copper, a droplet containing vesicles was dried, and excess solution is wiped using filter paper. After drying, the specimen is visualized as a small hollow vesicle with surrounding darkness (59, 57).

*Fourier Transforms Infrared Spectroscopy and Differential Scanning Calorimetry*

A differential calorimetry scanning study was carried out to examine the various lipid vesicles’ thermal behavior. An infrared spectroscopy study is used to determine any interactions among the components of the vesicle membrane and the drug (60).

*Entrapment Efficiency*

 The percentage entrapment of the drug added is called an entrapment efficiency. The entrapment efficiency was determined by the disruption of the vesicles after the separation of the non-entrapped drug. The percentage ratio of the concentration to the drug trapped to the total concentration of the drug is called an entrapment efficiency (61). As per this study (60), the vesicular suspension is centrifuged with a cooling centrifuge. The sediment was lysed with methanol after centrifugation. The absorbance was then measured in a UV-Visible spectrophotometer (62).

EE% = (ED/TD) × 100

ED is the drug concentration and TD is the total concentration of the drug.

*Degree of deformability*

The degree of deformity is a unique and important parameter of deformable vesicular formulation. It differs from other vesicular carriers like liposomes, which cannot extend across the intact stratum corneum. The relative deformity of the vesicles was determined using the extruder. The vesicular suspension was passed through the extruder containing polycarbonate filters of different pore sizes in the range of 50-200 nm. After extrusion, size distribution and the vesicle measurement were monitored using Malvern zeta sizer by the Dynamic light scattering method (63). The deformability value D is calculated by using the formula**, **

D = J (rv/rp)^2^

J = weight of the suspension which is extruded through the polycarbonate filters 

rv = vesicle size after extrusion, rp = barrier pore size.

*Number of vesicles per cubic mm*

The number of vesicles/cubic mm is the most important optimization parameter for composition and other process variables. Deformable vesicles can be counted using a hemocytometer with optical microscopy (13).

Total no. of vesicles per cubic mm =

 [Number of counted vesicles × dilution factor × 4000]/[Total number of counted Squares] 

*Stability studies*

 Technically, stability is defined as the capability of a specific formulation in a specific container/closure system-, to remain within its physical, chemical and therapeutic specification (64). Both the physical and chemical stability of the drug was evaluated. The physical stability has been assessed by visual observation for sedimentation and determination of particle size. The chemical stability was determined by the drug content measurement (65). 

*In-vitro diffusion study*

*In-vitro* diffusion study of elastic liposomes was evaluated using Franz diffusion cell apparatus. A cellophane membrane was pre-soaked for 24 h in phosphate buffer before being placed in between donor and receptor compartments. Elastic liposome formulation is placed on the sigma membrane in a donor compartment. The receptor compartment was filled with the required volume of phosphate buffer and stirred with a magnetic stirrer. The aliquots of the sample are withdrawn from the receptor compartment at definite time intervals and immediately replaced with an equal volume of fresh phosphate buffer solution to ensure sink condition. All the sample was analyzed using UV-Visible spectrophotometer (66).

*Skin penetration study*

 Franz diffusion cell was used for the skin penetration study. Different animals’ skin is used for skin penetration study example; fresh rat skin, porcine skin (57). The dorsal surface of the skin is mounted on a donor chamber containing vesicular formulation. The receptor compartment was filled with saline phosphate buffer solution and kept under stirring and maintained a temperature of 37 ± 0.5 °C for 24 h by circulating water through the external jacket of the cell. Aliquots of the sample under different time intervals are withdrawn and replaced with fresh buffer solution and analyzed using the suitable analytical method (60).

*Confocal Laser Scanning Microscopy Study (CSLM)*

CLSM was used to elucidate the penetration of elastic liposomal formulations through the skin. Formulations were loaded with Rhodamine 6G fluorescent probe (Rh6G, red). The fluorescence signal of Rh6G - loaded nanovesicles was scanned on different skin layers. A skin penetration study has to be carried out before performing the CLSM study. Rhodamine 6G - labeled vesicle was applied to the skin mounted on Franz cells and after 8hr of treatment, the skin was gently washed and the treated area was cut. The skin was frozen at -60 °C and the sample was obtained by a mechanical vertical section at a thickness of 10 µm. The analysis was conducted using CLSM at an emission wavelength of 560 nm and an excitation wavelength of 543 nm for the Rhodamine 6G probe (57).

*Histopathological study*

The histopathological analysis was carried out to determine the histopathological change of the skin after applying elastic liposome formulations. After the post-application of formulations on the skin, the treated area of the rat skin was excised and placed in a 10% formalin solution and sectioned vertically at 10 µm. The section was mounted on glass slides and stained with eosin and hematoxylin (E and H) and then examined by light microscopy (57).

**Future Remarks**

This article focused reviewing different deformable liposomes such as transfersomes, ethosomes, transethosomes, menthosomes and invasomes in the management of various diseases and its applications and importance. Researchers have found and realized the potential applications of deformable liposomes wherein improved permeation with deformability characteristics, entrapment efficiency, and reduced side effects compared THE other conventional liposomes. Despite its advantages with various applications, it faces certain limitations *i.e., it lacks several clinical and preclinical data required to accomplish the safety profile of newer developing drugs in the industrial sector and* based on its higher benefit and lower risk profile ratios. Researchers need to focus on the storage profile of deformable liposomes. The flexibility and deformability properties of deformable liposomes during prolonged storage, lead to less stability and lose their drug content, which hinder the scaling process and development of new dosage forms and oxidation of lipids also alters the storage of vesicular system (7, 67).

**Table 1:** Formulation components of deformable liposomes

**Deformable vesicles**	**Components**	**Examples**	**Uses**
Transfersomes	Edge activator (Surfactants)	Sodium deoxycholate, Sodium cholate, Spans, and Tweens, dipotassium glycyrrhizinate.	Increase the flexibility and elasticity of the lipid bilayer. (42)
	Phospholipids	(Soya phosphatidylcholine, egg phosphatidylcholine, Dipalmitoylphosphatidylcholine, *etc.*)	Membrane forming agent (56).
	Alcohol	Methanol, Ethanol,	solvent
	Hydrating medium	phosphate buffer of saline(pH 6.5-7)	buffering agent (43)
	Dye	Rhodamine 6G	For confocal laser scanning microscopy Study (52)
Ethosomes	Phospholipids	(Soya phosphatidylcholine, egg phosphatidylcholine, phosholipon^® ^90G, *etc.*)	Membrane forming agent (56)
	Alcohol	A higher concentration of Ethanol	As a Permeation enhancer (17)
	vehicle	Carbapol 934	Responsible for the gel’s mechanical strength (27)
	Dye	Nile red	For skin penetration study (18)
	cholesterol	cholesterol	For providing stability to the vesicle membrane, and entrapment efficiency of drugs
	Propylene glycol	Propylene glycol	Acts as a penetration enhancer (19)
Transethosomes	Edge activator	Tween 80, Span 60, Span 80, Span 65, Tween 20, Tween 60, sodium cholate, dipotassium glycyrrhizinate or sodium deoxycholate.	Increases the flexibility
	Phospholipids	Soya phosphatidylcholine and egg phosphatidylcholine.	Membrane forming agent
	Alcohol	A high amount of Ethanol	Enhances the skin permeation
	Permeation enhancers	Oleic acid	Increases the fluidity of the vesicles
	Buffer	Saline phosphate buffer pH 7.4	As a hydrating agent (39)
Menthosomes	Edge activator(cationic surfactants)	Cetrimide, cetyl pyridinium chloride	Increases the deformability of the bilayer.
	Phospholipid	Phosphatidylcholine (PC) from soybean	Membrane forming agent
	cholesterol	Cholesterol	As a stabilizer
	Buffer	0.01 M acetate buffer solution (pH 5.5)	Hydrating agent
	Penetration enhancer	l-menthol	Increases the skin permeation (56)
Invasomes	Phospholipids	Phospholipon 90G, Phospholipon 90H	Bilayer forming agent
	Ethanol and terpenes	Ethanol β-Citronellene, Citral, (R)-( + )-Limonene and (1R)- ( −)-Fenchone	Acts as a penetration enhancer (20)

**Table 2:** Applications of deformable liposomes

**Deformable Liposomes**	**Drug**	**Applications**	**Inference**
Transfersomes	Insulin	Delivery of Insulin	The developed transfersomal gel of insulin was able to reduce increased blood glucose levels
	Hydrocortisone and dexamethasone	Delivery of Corticosteroids	Application of transfersomal corticosteroids fastens the action, onset of anti-edema effects and bioactivity without affecting the mechanical abrasion.
	Meloxicam	Delivery of NSAIDs	Helped in the reduction of particle size with enhanced skin penetration and found better than conventional liposomes.
	5-Fluorouracil	Delivery of Anticancer agents	It enhanced the *in-vitro* skin permeation and deposition of the drug to the deeper parts of the skin and was better than the marketed preparation.
	Retinyl palmitate	Delivery of vitamin A derivatives	It demonstrated the compatibility of the transfersomes formulation and found a successful candidate for the effective delivery of highly lipophilic drugs.
	Lidocaine	Delivery of Local Anesthetics	The optimized lidocaine-transfersome formulation showed sustained delivery of drug compared to that of free drug
Ethosomes	Voriconazole	Delivery of Antifungal agents	*In-vitro* antifungal activity of the optimal ethosomal preparation was tested against *Aspergillus flavus *and was found to be successful for the treatment of fungal infections.
	Valsartan	Delivery of Anti-hypertensive drug	Showed a better and prolonged anti-hypertensive effect in valsartan-treated transdermal ethosomes compared to oral valsartan suspension
	Testosterone	Delivery of oral testosterone	The *in-vivo* and *in-vitro* study results showed enhanced bioavailability and skin permeation in the ethosomal formulation loaded with the testosterone over the marketed transdermal patch.
	Thymoquinone	Delivery of Anticancer agent	It showed enhanced skin permeation and deposition of drug and demonstrated anticancer activity against several tumors
	Febuxostat	Treatment of Gout	The formulated gel showed maximum skin penetration and was successful. It was found to be an alternative mode of therapy other than oral administration of tablets.
Transethosomes	ketorolac tromethamine	Delivery of NSAIDs	It showed inhibition of edema and swelling in transethosomes encapsulated ketorolac tromethamine gel
	colchicine	Treatment of Gout	The amount of colchicine permeated (after 24 h) through the skin of rats from the transethosomal gel was significantly higher than the NE gel.
	Voriconazole	Delivery of Antifungal drugs	It showed higher elasticity, permeability and skin deposition due to the synergistic effect of ethanol in conjunction with ethanol, edge activator or permeation enhancer
	Propranolol hydrochloride	Delivery of Anti-hypertensive drug	The formulated transethosomes was considered as a promising carrier for the delivery of non-selective β-blocker drug transdermally
Menthosomes	Meloxicam	Delivery of NSAIDs	Due to the presence of cationic surfactants, it significantly improved penetration of meloxicam through the skin, elasticity, flux, and deposition of the drug
Invasomes	Isradipine	Delivery of Anti-hypertensive agent	It showed a reduction of blood pressure in deoxycorticosterone acetate induced hypertensive rats after the application of isradipine loaded invasomal transgel
	Dapsone	Treatment of Acne	It showed higher skin deposition with improved penetration of drugs percutaneously and are compared to conventional liposomes
	Curcumin	Delivery of Herbal formulation	The formulation containing terpenes showed increased permeation in the skin due to the effect of terpenes and ethanol.
	Avanafil	Treatment of Erectile Dysfunction	It showed maximum skin penetration and was a good candidate for the transdermal delivery of drugs through the skin.

## Conclusion

Liposomes are small microscopic vesicles that are used in drug delivery but are confined to the upper parts of the stratum corneum. Research works have advanced its liposomal technology towards elastic liposomes, *i.e., *first-generation and second generation. Therefore, this review provides comprehensive information on numerous types of deformable liposomes, their mechanism of action, preparation methods, applications, and advantages over conventional liposomes. Due to the presence of edge activator (transfersomes), ethanol (Ethosomes), terpenes (invasomes), menthol (menthosomes), and combination of both ethanol and edge activator (transethosomes), these vesicles exhibit deformable characteristics that improve the flexibility, penetration, and delivery of therapeutically active agents such as drugs and vaccines to deeper parts of the tissue thus overcomes the problems of conventional liposomes. Researchers have found that these delivery systems are one of the successful ways to deliver poorly permeable drugs.
